# Myocardial Hydatid Cyst in a Young Male Patient Who Feeds Pet at Home: A Case Report

**DOI:** 10.1155/2012/413815

**Published:** 2012-11-26

**Authors:** Sinan Demirtas, Celal Yavuz, İsmail Basyigit, Ugur Firat, Ahmet Caliskan

**Affiliations:** ^1^Department of Cardiovascular Surgery, Dicle University, 21280 Diyarbakır, Turkey; ^2^Department of Pathology, Dicle University, 21280 Diyarbakır, Turkey

## Abstract

Hydatid cysts are rarely asymptomatic depending on their location. Complaints may be only relevant due to secondary problems. This paper presents a case report of a myocardial hydatid cyst which was totally excised under cardiopulmonary bypass operation with median sternotomy. Patient was admitted to the hospital with complaint of atypical chest pain in pungent manner together with shortness of breath. Various methods are used for diagnosis. However, echocardiography and computerized tomography with contrast are usually enough for definitive diagnosis and for deciding the strategy of operation. Curative excision must be performed. Extra care should be provided during the operation in order to prevent contamination. Postoperative appropriate antibiotherapy must be administered. Environment should be changed, and infrastructure must be improved in order to prevent further recurrences.

## 1. Introduction

 Hydatid cyst is an endemic infestation disease in various regions in the world. A parasite named “*Echinococcus granulosus*” lives inside cats and dogs which are its natural hosts. Humans are infected mostly via pets. Human myocardial involvement is very rare (%0.5–2) [1]. Its total incidence significantly decreased recently after socioeconomical level improvement with more hygienic community and environment. Contamination routes are gradually diminished with improvement of infrastructure. 

## 2. Presentation of Our Case

**Figure 1:**
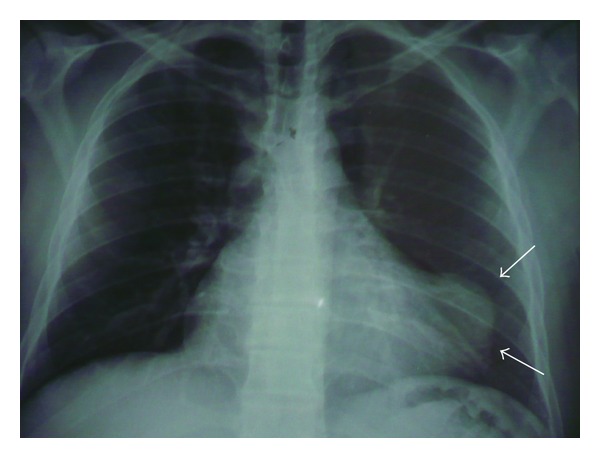
Preoperative teleradiography with apicolateral hydatid cyst image.

**Figure 2:**
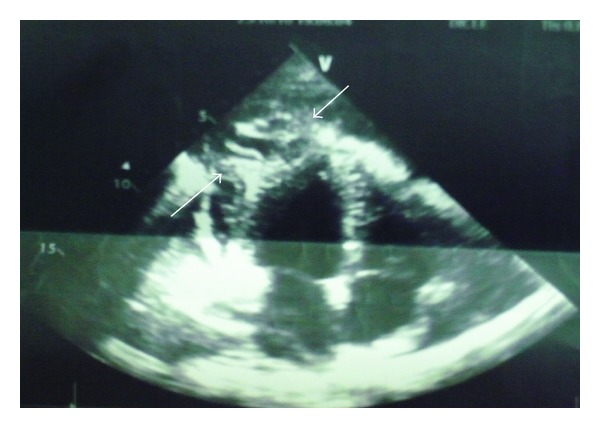
Preoperative echocardiography image. Lateral wall hydatid cyst image (arrows).

 24-year-old male patient had pungent-stinging chest pain with shortness of breath. His history revealed that he had many cats and dogs as pets in his house. His cardiovascular system examination revealed “decreased” heart sounds. Heart beats were coming from deep inside and barely heard. There were also barely heard respiratory sounds in his right lung and middle and basal segments. Other system examinations were normal. Vital signs were stable. There was no pathological finding in his electrocardiography. Teleradiography revealed enlarged mediastinum, closed right sinus, displacement of apex more superiorly and to the left side. When we looked more carefully, we noticed that extraspur at apex was not a solid mass. Instead, it was a kind of a radiolucent tissue (Figure 1). Transthoracic echocardiography revealed cystic structure with mobile-scolex inside. Diameter of cyst was 2.5 × 2.4 cm at the anterolateral section of left ventricle. Fibrinous pericardial effusion was also noticed within pericardial sac (Figure 2).

**Figure 3:**
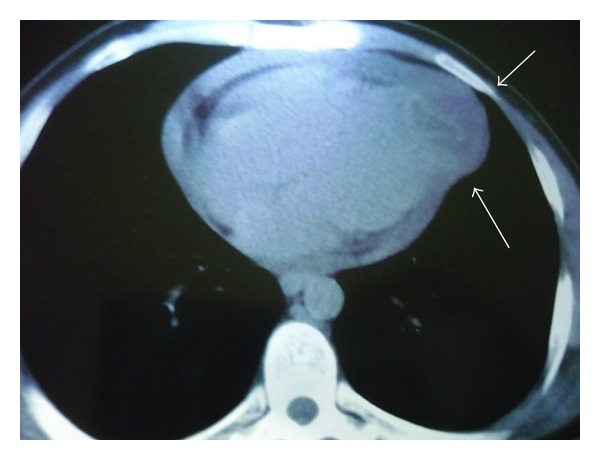
Preoperative contrast thorax CT image.

**Figure 4:**
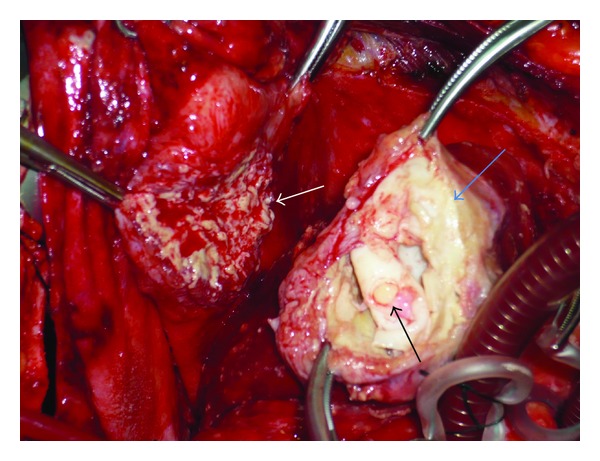
Image of cardiac cyst after dissection from adherent pericardium and opening of gauze pads with povidone-iodine. Pericardium, myocardium, cuticular membran, germinative, (blue arrow) membrane and daughter vesicles, (black arrow).

 Thorough examination of thoracoabdominal computerized tomography revealed 4.5 × 5 cm cystic mass inside heart apex, together with 2.5 cm pericardial effusion (Figure 3). There were no other cysts in lungs or liver. Final diagnosis was myocardial hydatid cyst. Treatment would be surgical so we decided our operative strategy. We performed median sternotomy and aortobicaval cannulation. During the operation, antegrade blood cardioplegic solution was administered. Systemic and topical cooling (with isotonic) was also performed. Approximately, 600 cc inflammatory and fibrinous pericardial effusion was aspirated. 1200 cc serous effusion was aspirated from right hemithorax. The cystic mass appeared to be limited between left anterior descending (LAD) artery and diagonal 2 arteries. It was not extending into cardiac cavities. It was also adherent or sticking to pericardium (Figure 4). For pathological examination and culture-antibiogram, the ingredients and content of cystical mass was aspirated with an injector. 7 cc purulent effusion was taken into injector. Then, we injected 8 cc hypertonic saline solution into cyst. We cautiously used green sterile covers with holes throughout the operation in order to prevent contamination. We used gauze-pads with povidone-iodine all around the operative site.

**Figure 5:**
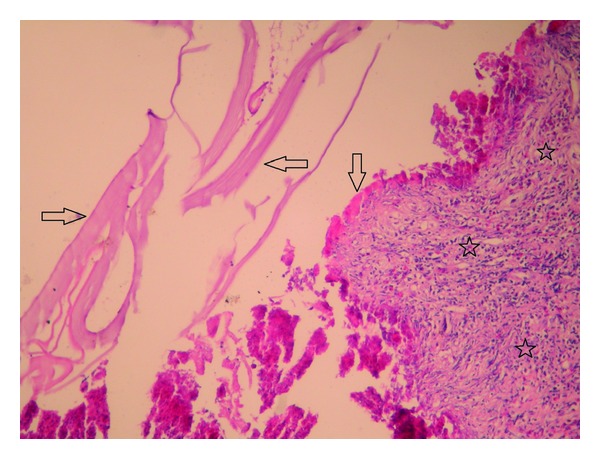
Hydatid cyst germinative membrane (right and left arrows), cuticular membrane (lower arrow), and inflammatory tissue rich of eosinophils (around stars) (H&E stain, ×100).

 We opened the cystic mass with oblique incision. We observed germinative membrane and 15 small-to-large daughter vesicles which were having different colors and shapes. We excised the cyst from myocardium together with its sticky capsule. Myocardial cavity was washed thoroughly with hypertonic saline solution. Then, we used teflon patch (felt) and sutured myocardial cavity using pledged-U-sutures. We protected the shape and appearance (geometry) of ventricle. Laboratory results revealed no reproduction at culture-antibiogram. Pathological examination confirmed the diagnosis of hydatid cyst both macroscopically and microscopically. Germinative membrane, cuticular membrane, and eosinophilic inflammation were detected (Figure 5). Preoperatively started (10 mg/kg/day) albendazol treatment was continued for 4 weeks even after the operation.

## 3. Discussion

Cardiac hydatid cyst produces symptoms depending on its location and surrounding structures. However, it may sometimes be completely asymptomatic or show up with only secondary complaints [2, 3]. 

 Our patient had shortness of breath and stinging chest pain. However, the symptoms of our patient were not related to myocardium itself; instead it was related to secondary problems which were pericardial effusion and adherent-sticky pericardium. 

 Today, treatment of cardiac cyst is surgical. Although many different methods are described in order to excise the cyst, the most preferred method is to inject hypertonic saline into the cyst itself and perform cystotomy. Cystectomy is performed after aspiration of contents; cyst is removed together with its adherent tissues [4, 5].

Other alternative methods include enucleation, especially left ventricle apical located cysts. After total excision, residual tissue may be closed with teflon patch similar to left ventricle aneurysmorhapy. Enucleation method is accepted to decrease recurrence; contour of heart is anatomically preserved after total removal of cyst [6, 7].

There are also some published data which suggest filling of residual cavity using biological glue [7]. Myocardial cystic mass excision has some complications like bleeding, harm to heart structures, rupture of cyst, and inoculation [8]. Under cardiopulmonary bypass, cyst should be covered with povidone-iodine absorbed gauze pads in order to decrease inoculation incidence. For this purpose, cyst contents are first aspirated and then hypertonic saline is injected before total excision. This method is safer. No residual tissue is left. Cardiac geometry should also be preserved [4]. 

 In conclusion, hydatid cyst may present itself with various symptoms. People with pets should be cautious and aware of this infestation.
